# One-year mortality of hematopoietic stem cell recipients admitted to an intensive care unit in a dedicated Brazilian cancer center: a retrospective cohort study

**DOI:** 10.1590/1516-3180.2021.0986.R1.11052022

**Published:** 2022-08-01

**Authors:** Leticia Vicentin Finencio Archanjo, Pedro Caruso, Antonio Paulo Nassar

**Affiliations:** I MSc. Nurse, Intensive Care Unit, A.C. Camargo Cancer Center, São Paulo (SP), Brazil.; II MD, PhD. Physician and ICU coordinator, Professor. A.C. Camargo Cancer Center, São Paulo (SP), Brazil. Professor, Discipline of Pulmonology, Universidade de São Paulo (USP), São Paulo (SP), Brazil.; III MD, PhD. Attending Physician and Professor, Intensive Care Unit, A.C. Camargo Cancer Center, São Paulo (SP) Brazil.

**Keywords:** Critical care, Hematopoietic stem cell transplantation, Mortality, Bone marrow transplantation, Renal replacement therapy, Hematological malignancy, Intensive care, Mechanical ventilation, Vasopressors

## Abstract

**BACKGROUND:**

Hematopoietic stem cell transplantation (HSCT) recipients requiring intensive care unit (ICU) admission early after transplantation have a poor prognosis. However, many studies have only focused on allogeneic HSCT recipients.

**OBJECTIVES:**

To describe the characteristics of HSCT recipients admitted to the ICU shortly after transplantation and assess differences in 1-year mortality between autologous and allogeneic HSCT recipients.

**DESIGN AND SETTING:**

A single-center retrospective cohort study in a cancer center in Brazil.

**METHODS:**

We included all consecutive patients who underwent HSCT less than a year before ICU admission between 2009 and 2018. We collected clinical and demographic data and assessed the 1-year mortality of all patients. The effect of allogeneic HSCT compared with autologous HSCT on 1-year mortality risk was evaluated in an unadjusted model and an adjusted Cox proportional hazard model for age and Sequential Organ Failure Assessment (SOFA) at admission.

**RESULTS:**

Of the 942 patients who underwent HSCT during the study period, 83 (8.8%) were included in the study (autologous HSCT = 57 [68.7%], allogeneic HSCT = 26 [31.3%]). At 1 year after ICU admission, 21 (36.8%) and 18 (69.2%) patients who underwent autologous and allogeneic HSCT, respectively, had died. Allogeneic HSCT was associated with increased 1-year mortality (unadjusted hazard ratio, HR = 2.79 [confidence interval, CI, 95%, 1.48–5.26]; adjusted HR = 2.62 [CI 95%, 1.29–5.31]).

**CONCLUSION:**

Allogeneic HSCT recipients admitted to the ICU had higher short- and long-term mortality rates than autologous HSCT recipients, even after adjusting for age and severity at ICU admission.

## INTRODUCTION

Hematopoietic stem cell transplantation (HSCT) is a potentially curative therapy for many hematological malignancies. HSCT is mainly classified as either autologous or allogeneic. Stem cells are obtained from the patients in autologous HSCT and related or unrelated donors in allogeneic HSCT. The most common indications for autologous HSCT are multiple myeloma and lymphomas. Allogeneic HSCT is commonly indicated for leukemia and myelodysplastic syndromes.^1^

HSCT may also cause life-threatening complications secondary to the conditioning regimen, engraftment, and posterior immunosuppression in the case of allogeneic HSCT, which may ultimately lead to intensive care unit (ICU) admission.^2,3^ Historically, HSCT recipients admitted to the ICU had grim prognoses. Nevertheless, outcomes have significantly improved during the past decades.^4,5^ However, outcomes in allogeneic HSCT recipients with ICU admission may have plateaued in the last 10 years.^6^

Previous studies have focused mainly on allogeneic HSCT recipients,^7^ been carried out in specific centers in high-income countries,^4,5^ and focused on short-term outcomes.^4,7^ Few studies have addressed the characteristics and outcomes of autologous HSCT recipients admitted to the ICU,^8,9,10^ in middle- or low-income countries,^11^ or focused on long-term outcomes.^12,13^

## OBJECTIVE

The present study aimed to describe a cohort of HSCT recipients admitted to the ICU in a dedicated Brazilian cancer center from 2009 to 2018 and assess differences in long-term mortality between autologous and allogeneic HSCT recipients admitted to the ICU shortly after HSCT.

## METHODS

### Design and setting

This retrospective cohort study was conducted at a dedicated reference center for HSCT in São Paulo, Brazil. The current database included all patients admitted between September 2009 and December 2018. The local institutional review board approved the study (CAAE 86761718.0.0000.5432; dated June 6, 2018) and waived the need for informed consent. We followed the recommendations of the STROBE statement, which guides the reporting of observational studies.^14^

### Participants

We included all consecutive patients who underwent HSCT less than a year before ICU admission during the study period. We only considered the first admission in patients admitted to the ICU more than once. We excluded patients younger than 18 years of age. We retrieved patient data from a local database and electronic medical records. We collected baseline data on age, sex, Eastern Cooperative Oncology Group (ECOG) performance status before ICU admission (registered by the intensivist in charge at the ICU admission, based on reports by family members or emergency department/rapid response team physician), comorbidities, Charlson Comorbidity Index (CCI) from the ICU admission chart, hematological malignancy ultimately leading to HSCT, type of HSCT (autologous or allogeneic), conditioning regimen, and graft-versus-host disease (GVHD) from the HSCT multidisciplinary chart. We also collected data on ICU admission: type of admission (medical or surgical), the reason for admission, and patient severity at admission (measured by the Simplified Acute Physiology Score [SAPS] 3).^15,16^ We calculated the Sequential Organ Failure Assessment (SOFA) score from days 1 to 7 after ICU admission, retrieving the vital signs and laboratory results from the medical chart. When laboratory results (i.e., bilirubin and creatinine) were considered normal.^17^ Additionally, we collected data on the use of organ support (vasopressor therapy, non-invasive and invasive mechanical ventilation, and renal replacement therapy), ICU and hospital outcomes (length of stay [LOS] and mortality), and 1-year mortality. We checked the medical records to identify patients’ last appointments. All patients were censored at this time point. We compared the characteristics; of ICU, hospital, and 1-year outcomes; and overall survival of autologous and allogeneic HSCT recipients.

### Statistical analysis

This study was mainly descriptive. We did not perform sample size or power calculations; instead, we presented all available data of the included patients. All data are presented as frequencies (percentages) for categorical variables and medians (interquartile range [IQR]) for continuous variables. We used the10.3747 Chi-square test of independence or Fisher’s exact test for categorical variables and the Mann–Whitney test for continuous variables to compare the two groups.

We used Kaplan–Meier plots and log-rank tests to analyze the differences in overall survival time between autologous and allogeneic HSCT recipients. We used the Cox proportional hazard regression model to assess the effect of allogeneic HSCT compared with autologous HSCT on 1-year mortality risk in an unadjusted model and an adjusted model for age and SOFA score at admission. The proportional hazard assumption for the models was verified using the Schoenfeld residuals method. We calculated this model’s hazard ratio (HR) and 95% confidence interval (CI 95%). We used R version 4.1.1 (R Core Team, Vienna, Austria, 2021) for all analyses with the following packages: survival, survminer, and ggplot2.

## RESULTS

**Figure 1.**
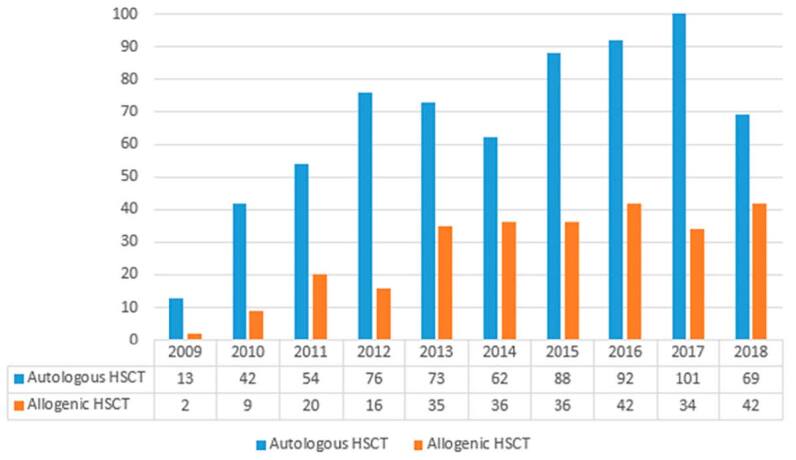
Total number of patients who underwent hematopoietic stem cell transplantation between September 2009 and December 2018.

**Figure 2.**
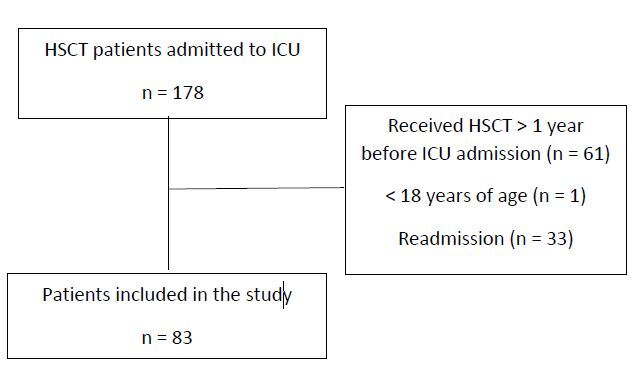
Study flowchart.

During the study period, 942 patients underwent HSCT (autologous, n = 670 [71.1%]; allogeneic, n = 272 [28.9%]) (Figure 1). There were 178 admissions of patients to the ICU up to 1 year after HSCT. Of these, 83 patients were included in the study (Figure 2). The median time from HSCT to ICU admission was 12 (IQR, 7–94) days.

HSCT = hematopoietic stem cell transplant.

HSCT = hematopoietic stem cell transplant; ICU = intensive care unit.

Acute leukemia was the most common malignancy necessitating allogeneic HSCT. Multiple myeloma and lymphoma were the most common malignancies leading to autologous HSCT. Allogeneic HSCT recipients were younger, more commonly admitted due to acute respiratory failure, and more frequently required mechanical ventilation than autologous HSCT recipients. Sepsis was the most common reason for admission for autologous HSCT. The SAPS 3 and SOFA scores at admission were not different between autologous and allogeneic HSCT recipients. However, allogeneic HSCT recipients had higher ICU and hospital mortality rates (Table 1).

**Table 1.** Characteristics and outcomes of hematopoietic stem cell transplant (HSCT) recipients admitted to the intensive care unit

	Autologous HSCT (n = 57)	Allogeneic HSCT (n = 26)	P
**Gender**			0.47
Female	28 (49.1)	15 (57.7)	
Male	29 (50.9)	11 (42.3)	
**Age**	57.8 (45.6–62.4)	43.2 (24.7–58.2)	< 0.01
**Comorbidities**			
Arterial hypertension	36 (63.2)	7 (26.9)	0.38
Diabetes mellitus	8 (14.0)	4 (15.4)	0.87
Heart failure	7 (12.3)	1 (3.8)	0.31
Coronary artery disease	5 (8.8)	0	0.12
Peripheral vascular disease	4 (7.0)	0	0.17
Chronic obstructive pulmonary disease	6 (10.5)	1 (3.8)	0.31
Chronic kidney disease	5 (8.8)	2 (7.7)	0.87
**CCI**	2 (2–3)	2 (2–3)	0.38
**Hematological malignancy**			< 0.01
Multiple myeloma	27 (47.4)	1 (3.8)	
Non-Hodgkin Lymphoma	13 (22.8)	5 (19.2)	
Hodgkin Lymphoma	16 (28.1)	2 (7.7)	
Acute lymphocytic leukemia	0	7 (26.9)	
Acute myeloid leukemia	0	7 (26.9)	
Chronic myeloid leukemia	0	2 (7.7)	
Other hematological malignancies	1 (1.8)	2 (7.7)	
**ECOG**			0.44
0	19 (33.3)	4 (15.4)	
1	14 (24.6)	10 (38.5)	
2	12 (21.1)	5 (19.2)	
3	4 (7.0)	3 (11.5)	
4	8 (14.0)	4 (15.4)	
**Source of admission**			0.59
Wards	47 (82.5)	19 (73.1)	
Emergency room	9 (15.8)	6 (23.1)	
Surgical room	1 (1.8)	1 (3.8)	
**Type of admission**			0.67
Medical	55 (96.5)	25 (96.1)	
Surgical	2 (3.5)	1 (3.9)	
**Reason for admission**			< 0.01
Sepsis	27 (47.4)	7 (26.9)	
Acute respiratory failure	16 (28.1)	12 (46.2)	
Cardiovascular	12 (21.1)	1 (3.8)	
Neurological	5 (8.8)	5 (19.2)	
Acute kidney injury	3 (5.3)	1 (3.8)	
**SAPS 3**	82 (68–86)	75 (64.5–82.5)	0.16
**SOFA**	5 (3.5–6)	5 (3–6)	0.68
**ICU Organ support**			
Vasopressors	22 (38.6)	13 (50.0)	0.68
Non-invasive mechanical ventilation	16 (28.1)	11 (42.3)	0.32
Invasive mechanical ventilation	12 (21.1)	13 (50.0)	0.01
Renal replacement therapy	7 (12.3)	5 (19.2)	0.60
**ICU mortality**	7 (12.3)	10 (38.5)	< 0.01
**ICU LOS**	3 (2–7)	4.5 (1–11.25)	0.62
**Hospital mortality**	12 (21.1)	15 (57.7)	< 0.01
**Hospital LOS**	19 (13–26)	20.5 (11–42.5)	0.51

CCI = Charlson Comorbidity Index; ECOG = Eastern Cooperative Oncology Group; ICU = intensive care unit; LOS = length of stay; SAPS 3 = Simplified Acute Physiology Score 3.

Among allogeneic HSCT recipients, 19 patients (73.1%) received a myeloablative conditioning regimen, and seven (26.9%) received a non-myeloablative conditioning regimen. Only six (23.1%) patients received stem cells from an unrelated donor. A total of 16 patients had GVHD (61.5%): 13 (81.2%) had skin involvement; eight (50%), gastrointestinal; three (18.7%), lung; and two (12.5%), liver.

**Figure 3.**
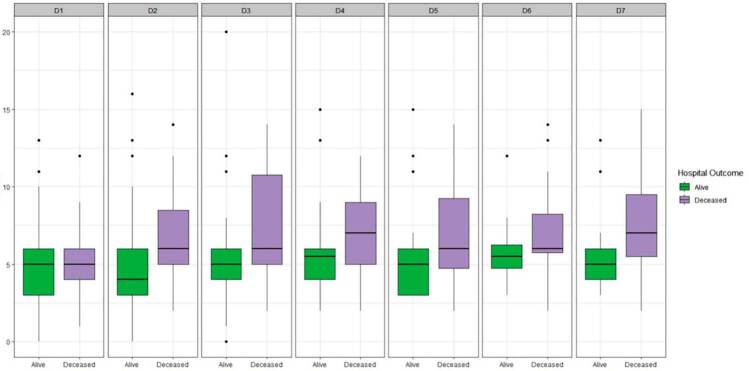
Sequential Organ Failure Assessment (SOFA) from days 1 to 7 in alive and deceased patients at hospital discharge.

Although not different at admission, HSCT recipients who ultimately died at hospital discharge had increased SOFA scores 2 to 7 days after ICU admission (Figure 3).

**Figure 4.**
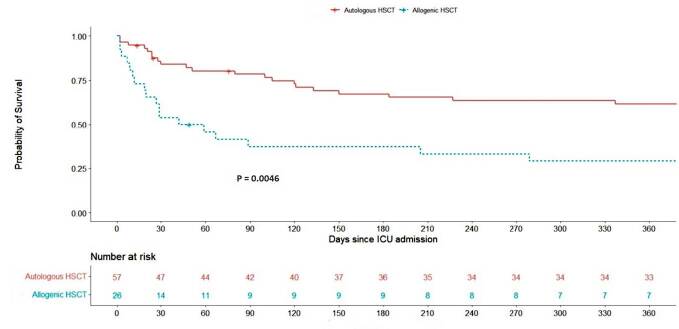
Survival of autologous and allogeneic hematopoietic stem cell recipients admitted to the intensive care unit.

After ICU admission, we followed up the patients for a median of 279 (IQR, 29–1670) days. Median survival after ICU admission was 50.5 (CI 95%, 20–430) days for allogeneic HSCT recipients and 1115 (CI 95%, 337–NA) days for autologous HSCT recipients. At 1 year after ICU admission, 21 (36.8%) autologous HSCT recipients and 18 (69.2%) allogeneic HSCT recipients had died (Figure 4). Allogeneic HSCT was associated with an increased 1-year mortality (unadjusted HR = 2.79 [CI 95%, 1.48–5.26]; adjusted HR = 2.62 [CI 95%, 1.29–5.31]).

HSCT = hematopoietic stem cell transplant; ICU = intensive care unit.

## DISCUSSION

Almost 9% of the HSCT recipients were admitted to the ICU during the study period. As expected, allogeneic HSCT recipients were younger and had acute leukemia as the primary hematological cancer. Autologous HSCT recipients were older and had mainly multiple myeloma and lymphoma as baseline malignancies. Although not more severely ill at ICU admission, allogeneic HSCT recipients had higher short-term mortality rates than autologous HSCT recipients. Allogeneic HSCT was also associated with 1-year mortality, even after adjusting for age and severity of organ dysfunction at admission.

Previous studies have found that approximately 3%–10% of autologous HSCT recipients^8,10,18^ and 15%–25% of allogeneic HSCT recipients require ICU admission after the first few months of transplantation. ICU and hospital mortality in allogeneic HSCT recipients admitted to the ICU ranges from 40% to 90%.^4,12,19,20^ Small cohorts of autologous HSCT recipients had ICU mortality rates from 20 to 60%.^8,10^ We found similar mortality rates in both autologous and allogeneic HSCT recipients to those reported in studies carried out in high-income countries. In contrast, a Mexican study of 17 autologous and 51 allogeneic HSCT recipients who required ICU admission during a 20-year period found that 88% and 90% of the patients died at ICU discharge, respectively,.^21^

The severity of organ dysfunction has consistently been associated with worse outcomes in critically ill HSCT recipients.^20^ Our data suggested that patients who did not survive to hospital discharge had worsening organ dysfunction during the first days after ICU admission. The requirement for mechanical ventilation is an important predictor of mortality in allogeneic HSCT recipients. Studies that included allogeneic and autologous HSCT recipients have also suggested high mortality rates for patients requiring mechanical ventilation.^18,22,23^ In our study, although the initial severity measured by SAPS 3 and SOFA scores did not differ between autologous and allogeneic HSCT recipients, 50% of allogeneic HSCT recipients required invasive mechanical ventilation during their ICU stay. In comparison, it only occurred in 21% of autologous HSCT recipients. The severity of respiratory failure after ICU admission may be responsible for our study’s higher ICU and hospital mortality rates found in allogeneic HSCT recipients.

Few studies have addressed the long-term outcomes of HSCT recipients admitted to the ICU. Approximately 70% to 80% of all allogeneic HSCT recipients admitted to the ICU shortly after the transplant die within 1 year.^5,12,19^ The median overall survival may be as poor as 41 days.^24^ Our findings of a 1-year mortality rate of 69% and median survival of 50 days are similar to those of previous studies. A French study of 27 autologous HSCT recipients admitted to the ICU showed a 6-month mortality rate of 27%.^8^ On the other hand, a study with data from 1992 to 2002 in Ontario, Canada, showed a 1-year mortality rate of 70% for autologous HSCT recipients admitted to ICU.^18^ Another Canadian study of 34 autologous HSCT recipients showed a mean survival of almost 29 months.^10^ In our study, we found a mortality rate of 36.8% at 1 year and a median survival of more than 3 years.

Allogeneic HSCT was associated with higher 1-year mortality, even when adjusted for age and severity of organ dysfunction at ICU admission. In addition, a previous study had shown that allogeneic HSCT was associated with an increased risk of mortality 6 months after ICU admission in an adjusted Cox proportional hazards model, which also included the requirement for mechanical ventilation and vasopressor during ICU stay.^22^ Probably, the severity of the hematological baseline condition that led to the transplant and specific characteristics of the allogeneic HSCT, such as the requirement for immunosuppression and GVHD occurrence, may have a negative impact on the survival of allogeneic HSCT recipients admitted to the ICU.

Our study had some limitations. First, it was a single-center study. Therefore, our findings may not be generalizable to other settings. However, this has been a common limitation in most studies addressing critically ill HSCT recipients, as only a few were multicenter studies. Second, the small sample size precluded further analysis of the impact of other variables, such as GVHD or conditioning regimens, on long-term mortality. Therefore, our study should be considered descriptive. Third, retrospective studies are prone to information bias, and some useful information may have been inadequately described in medical charts.

## CONCLUSION

In conclusion, almost 9% of all patients who underwent HSCT were admitted to the ICU within 1 year of the transplantation. Allogeneic HSCT recipients had higher short- and long-term mortality rates than autologous HSCT recipients, even after adjusting for age and severity at ICU admission. Worsening organ dysfunction in the first days after ICU admission in HSCT recipients should be considered to establish realistic goals of care for these patients.

## References

[1] Bazinet A, Popradi G (2019). A general practitioner’s guide to hematopoietic stem-cell
transplantation. Curr Oncol..

[2] Fornwalt RA, Brigham EP, Scott Stephens R (2021). Critical Care of Hematopoietic Stem Cell Transplant
Patients. Crit Care Clin..

[3] Lengliné E, Mirouse A, Azoulay E (2019). Top ten tips for the management of critically ill hematopoietic
stem cell transplantation recipients. Intensive Care Med..

[4] Lengliné E, Chevret S, Moreau AS (2015). Changes in intensive care for allogeneic hematopoietic stem cell
transplant recipients. Bone Marrow Transplantation..

[5] Lueck C, Stadler M, Koenecke C (2018). Improved short- and long-term outcome of allogeneic stem cell
recipients admitted to the intensive care unit: a retrospective longitudinal
analysis of 942 patients. Intensive Care Med..

[6] Darmon M, Bourmaud A, Georges Q (2019). Changes in critically ill cancer patients’ short-term outcome
over the last decades: results of systematic review with meta-analysis on
individual data. Intensive Care Med..

[7] Saillard C, Darmon M, Bisbal M (2018). Critically ill allogenic HSCT patients in the intensive care
unit: a systematic review and meta-analysis of prognostic factors of
mortality. Bone Marrow Transplantat..

[8] Kerhuel L, Amorim S, Azoulay E, Thiéblemont C, Canet E (2015). Clinical features of life-threatening complications following
autologous stem cell transplantation in patients with
lymphoma. Leuk Lymphoma..

[9] Jenkins P, Johnston LJ, Pickham D (2015). Intensive Care Utilization for Hematopoietic Cell Transplant
Recipients. Biol Blood Marrow Transplant..

[10] Trinkaus MA, Lapinsky SE, Crump M (2009). Predictors of mortality in patients undergoing autologous
hematopoietic cell transplantation admitted to the intensive care
unit. Bone Marrow Transplantat..

[11] Barreto LM, Torga JP, Coelho SV, Nobre V (2015). Main characteristics observed in patients with hematologic
diseases admitted to an intensive care unit of a Brazilian university
hospital. Rev Bras Ter Intensiva..

[12] Platon L, Amigues L, Ceballos P (2016). A reappraisal of ICU and long-term outcome of allogeneic
hematopoietic stem cell transplantation patients and reassessment of
prognosis factors: results of a 5-year cohort study
(2009-2013). Bone Marrow Transplant..

[13] Mokart D, Lambert J, Schnell D (2013). Delayed intensive care unit admission is associated with
increased mortality in patients with cancer with acute respiratory
failure. Leuk Lymphoma..

[14] von Elm E, Altman DG, Egger M (2008). The Strengthening the Reporting of Observational Studies in
Epidemiology (STROBE) statement: guidelines for reporting observational
studies. J Clin Epidemiol..

[15] Metnitz PG, Moreno RP, Almeida E (2005). SAPS 3--From evaluation of the patient to evaluation of the
intensive care unit. Part 1: Objectives, methods and cohort
description. Intensive Care Med..

[16] Moreno RP, Metnitz PG, Almeida E (2005). SAPS 3--From evaluation of the patient to evaluation of the
intensive care unit. Part 2: Development of a prognostic model for hospital
mortality at ICU admission. Intensive Care Med..

[17] Vincent JL, Moreno R, Takala J (1996). The SOFA (Sepsis-related Organ Failure Assessment) score to
describe organ dysfunction/failure. On behalf of the Working Group on
Sepsis-Related Problems of the European Society of Intensive Care
Medicine. Intensive Care Med..

[18] Scales DC, Thiruchelvam D, Kiss A, Sibbald WJ, Redelmeier DA (2008). Intensive care outcomes in bone marrow transplant recipients: a
population-based cohort analysis. Crit Care..

[19] Mokart D, Granata A, Crocchiolo R (2015). Allogeneic hematopoietic stem cell transplantation after reduced
intensity conditioning regimen: Outcomes of patients admitted to intensive
care unit. J Crit Care..

[20] Orvain C, Beloncle F, Hamel JF (2018). Allogeneic stem cell transplantation recipients requiring
intensive care: time is of the essence. Ann Hematol..

[21] Galindo-Becerra S, Labastida-Mercado N, Rosales-Padrón J (2015). Outcome of Recipients of Hematopoietic Stem Cell Transplants Who
Require Intensive Care Unit Support: A Single Institution
Experience. Acta Haematol..

[22] Huynh TN, Weigt SS, Belperio JA, Territo M, Keane MP (2009). Outcome and prognostic indicators of patients with hematopoietic
stem cell transplants admitted to the intensive care unit. J Transplant..

[23] Yadav H, Nolan ME, Bohman JK (2016). Epidemiology of Acute Respiratory Distress Syndrome Following
Hematopoietic Stem Cell Transplantation. Crit Care Med..

[24] Nakamura M, Fujii N, Shimizu K (2018). Long-term outcomes in patients treated in the intensive care unit
after hematopoietic stem cell transplantation. Int J Hematol..

